# Toileting behaviors and overactive bladder in patients with type 2 diabetes: a cross-sectional study in China

**DOI:** 10.1186/s12894-017-0234-2

**Published:** 2017-06-13

**Authors:** Dongjuan Xu, Ran Cheng, Aixia Ma, Meng Zhao, Kefang Wang

**Affiliations:** 1 School of Nursing, Shandong University, No.44, Wenhua Xi Road, Jinan, Shandong 250012 China; 2 School of Nursing, Purdue University, West Lafayette, Indiana 47907 USA; 3 Department of endocrinology, Qilu Hospital of Shandong University, Jinan, Shandong 250012 China

**Keywords:** Overactive bladder, Diabetes mellitus, Toileting behaviors

## Abstract

**Background:**

Overactive bladder is more prevalent in patients with type 2 diabetes than in those without diabetes. Unhealthy toileting behaviors may be associated with the development and worsening of overactive bladder symptoms. However, little is known about the relationships between toileting behaviors and overactive bladder in patients with diabetes. This study aimed to identify unhealthy toileting behaviors that patients with type 2 diabetes adopted to empty their bladders and investigate the relationships between toileting behaviors and overactive bladder.

**Methods:**

Patients with type 2 diabetes from the endocrinology outpatient department of a hospital in China were recruited. The Toileting Behaviors-Women’s Elimination Behavior and Overactive Bladder Symptom Score questionnaires were used to assess the patients’ toileting behaviors and overactive bladder symptoms. A multivariate logistic regression model was used to explore the relationships between toileting behaviors and overactive bladder.

**Results:**

Almost 14% of patients with diabetes had overactive bladder. The unhealthiest toileting behavior was premature voiding. In the multivariate logistic regression analysis, premature voiding (OR = 1.286, *p* = 0.016) and straining to void (OR = 1.243, *p* = 0.026) were associated with overactive bladder. There was a greater likelihood of having overactive bladder when patients engaged in unhealthy toileting behaviors (premature voiding and straining to void).

**Conclusions:**

Overactive bladder in patients with type 2 diabetes was more than twofold higher than that in the general population. Thus, overactive bladder is not just an inconsequential condition for patients with diabetes. Unhealthy toileting behaviors, e.g., premature voiding and straining to void, may contribute to the onset or worsening of overactive bladder in patients with diabetes. Identification and awareness of these modifiable behavioral factors during diabetes care is an essential component of primary prevention, alleviation, and management of overactive bladder symptoms.

## Background

Overactive bladder, defined as “urinary urgency, usually accompanied by frequency and nocturia, with or without urgency urinary incontinence” [1], affects a large proportion of the general adult population. Approximately 455 million people worldwide (almost 11%) experience overactive bladder, and the regional burden of overactive bladder is estimated to be greatest in Asia [2]. A recent large population-based survey conducted in China found that the prevalence of overactive bladder was 6% [3]. Overactive bladder can have a substantial negative effect on patients’ quality of life specifically in terms of physical and psychological well-being, social interactions, work productivity, and sexual health [4, 5]. In addition, the direct and indirect costs of overactive bladder could seriously aggravate the economic burden on individuals and healthcare systems [6].

Unhealthy toileting behaviors may be associated with the development and worsening of overactive bladder symptoms. Individuals may empty bladder more frequently than necessary, based on the misconception that frequent voiding avoids bladder incontinence episodes [7]. However, too frequent voiding only signal an urge to empty the bladder despite a smaller volume of urine [8], which can precipitate or exacerbate bladder dysfunction [9]. Straining to empty the bladder, which involves an abdominal muscle contraction, could increase the peak flow and mean flow rates, as well as decrease the total voiding time [10]. Individuals who use straining to start voiding before the initiation of the micturition reflex may be more likely to develop incontinence and voiding dysfunction [11]. Toileting behavior is a comprehensive concept that includes voiding place, time, position, and style [12]; however, most recent studies on toileting behavior and overactive bladder focused only on one or some aspects of toileting behavior [13, 14]. Previously, Wan et al. examined the relationship between toileting behaviors and lower urinary tract symptoms among female nurses and found that three unhealthy toileting behaviors (i.e., premature voiding, delayed voiding, and straining to void) were significantly associated with lower urinary tract symptoms, while no significant association with voiding place or position preference was observed [15]. However, little is known about the relationships between toileting behaviors and overactive bladder in other populations.

Diabetes mellitus has been related to an earlier onset and increased bladder dysfunction severity [16]. Overactive bladder is more prevalent in patients with type 2 diabetes than in the general population [17, 18]. Moreover, higher glycosylated hemoglobin levels increased the risk of overactive bladder in patients with diabetes [19]. Besides diabetes-related factors, a variety of factors were associated with overactive bladder, including age [3, 19], gender [20], marital status [3], fluid intake [21], smoking [22], alcohol drinking [3], physical exercise [23], history of urinary tract infection [24], constipation [25], hypertension/heart failure [26], and obesity [20].

Because of its high prevalence, overactive bladder is an important therapeutic target in patients with type 2 diabetes. Available evidence suggests that behavioral interventions, such as bladder training [27], pelvic floor muscle training and exercise, and urge suppression techniques [28], are effective in extending voiding interval and alleviating urgency and incontinence. Behavioral interventions may also increase the efficacy of pharmacologic treatment for overactive bladder [9]. If unhealthy toileting behaviors can contribute to overactive bladder symptoms, identifying and modifying these unhealthy behaviors, accompanied by other behavioral and pharmacologic treatments, help prevent, eliminate, alleviate, and manage overactive bladder symptoms in patients with type 2 diabetes. Therefore, the purposes of this study were (1) to investigate the unhealthy toileting behaviors that patients with diabetes adopted to empty their bladders and (2) to identify the relationships between toileting behaviors and overactive bladder in patients with type 2 diabetes.

## Methods

### Study design and data collection

This is a cross-sectional study with convenience sampling. From May to August 2014, we recruited participants from the endocrinology outpatient department in one of the largest hospitals in Jinan, the capital city of Shandong Province, China. The inclusion criteria were as follows: (1) ≥18 years old, (2) diagnosed as having type 2 diabetes, and (3) willing to participate in the study and capable of understanding study procedure and questions. The exclusion criteria were the following: (1) new-onset neurological disorders (such as stroke, spinal cord damage, Parkinson’s disease, and multiple sclerosis), (2) urinary tract infections within the last month of the survey, (3) pelvic organ prolapse, and (4) history of bladder surgery.

The Institutional Review Board of Shandong University approved this study. A self-administered pencil-and-paper survey was used to collect data. Before the survey, trained graduate students obtained written informed consent from each patient with diabetes. The survey was completed anonymously and the patients were assured that their responses would be kept confidential.

### Assessment

We obtained the patients’ sociodemographic characteristics data, including age, sex, race/ethnicity, education, marital status, living area, and income, and assessed lifestyle-related characteristics, including smoking, alcohol use, tea drinking, fluid intake, and physical exercise. We also collected health-related data consisting of waist circumference, hip circumference, urinary tract infection history, comorbidity, diabetes mellitus duration, and microvascular complications of diabetes (peripheral neuropathy and retinopathy). Moreover, the Charlson comorbidity index (CCI) was used to measure a range of comorbid conditions, such as hypertension, congestive heart failure, or cancer (a total of 20 conditions) [29]. Since all patients in this study had diabetes, diabetes was excluded from the calculation of CCI. Each condition was assigned a weight of 1, 2, 3, or 6, depending on the risk of mortality [29]. The total score, which is the sum of all weighted conditions, was used to predict mortality.

We used the validated Toileting Behaviors-Women’s Elimination Behavior (TB-WEB) scale (Chinese version) to evaluate the participants’ toileting behaviors [30]. This 17-item scale contains five domains: place preference for voiding, premature voiding, delayed voiding, straining to void, and position preference for voiding. Since position preference for voiding (e.g., “crouching or hovering to empty bladder when not at home”) is specific for the female population, we did not include this domain in the study. Detailed descriptions of each item are provided in Table 3. The TB-WEB used a five-point Likert-type scale (from 1 = never to 5 = always) to assess how often patients with diabetes adopted a behavior. An average score was calculated for each domain, and a higher score corresponding to unhealthier toileting behavior. The 4-domain TB-WEB has been validated in male patients [31], and the Cronbach’s alpha coefficient of the TB-WEB scale was 0.71 in this study.

We used the Chinese version of Overactive Bladder Symptom Score (OABSS) questionnaire to evaluate overactive bladder symptoms in the past week. OABSS is a reliable and valid questionnaire designed to quantify the four symptoms of overactive bladder: daytime frequency (0–2), nighttime frequency (0–3), urgency (0–5), and urgency incontinence (0–5) [32]. The total score ranges from 0 to 15, and a higher score represents more severe overactive bladder symptoms. The Chinese version of the OABSS scale has been developed and validated in the Chinese population with good reliability and validity [33]. Here, overactive bladder means an urgency symptom score ≥ 2 and a total score ≥ 3.

### Statistical analysis

We used descriptive statistics (frequency and percentage for categorical variables, mean and standard deviation for continuous variables) to describe the characteristics of patients with type 2 diabetes. We performed Student’s *t*-tests to compare the differences of each toileting behavior between patients without overactive bladder and those with overactive bladder. We also used a multivariate logistic regression model to explore the relationships between toileting behaviors and overactive bladder, after adjusting for age, sex, race/ethnicity, education, marital status, living area, income, smoking, alcohol use, tea drinking, fluid intake per day, physical exercise, waist-to-hip ratio, urinary tract infection history, CCI, diabetes mellitus duration, and microvascular complications of diabetes (peripheral neuropathy and retinopathy). We performed all statistical analyses using Stata (Version 14.1; StataCorp, College Station, TX). Statistical significance was accepted at *p* < 0.05.

## Results

Among the 1025 eligible patients with type 2 diabetes, seven patients were excluded from the analysis because they omitted four or more survey items. The mean age was approximately 59 years (range 20–86 years), and half of the participants were female. The patients had diabetes for about 9 years on average, and more than half (52%) had diabetic peripheral neuropathy or retinopathy. Nearly 14% of the patients had overactive bladder. Among the overactive bladder patients, approximately 57% had wet overactive bladder and 43% had dry overactive bladder. The patient characteristics are presented in Table 1.

**Table 1:** Characteristics of patients with type 2 diabetes mellitus (*n* = 1018)

Variables	Mean ± SD or *n* (%)
Age (years)	59.1 ± 11.7
Sex	
Female	509 (50.0)
Male	509 (50.0)
Race/ethnicity	
Han	981 (96.4)
Other	37 (3.6)
Education	
Elementary school or lower	228 (22.4)
Middle school	232 (22.8)
High school	306 (30.1)
College or higher	252 (24.7)
Marital status	
Married	920 (90.4)
Single/divorced/widowed	98 (9.6)
Living area	
Urban	770 (75.6)
Rural	248 (24.4)
Income (RMB (USD)/month)	
≤3000 (451)	620 (60.9)
>3000 (451)	398 (39.1)
Smoker	
Yes	345 (33.9)
No	673 (66.1)
Alcohol drinker	
Yes	308 (30.3)
No	710 (69.7)
Tea drinker	
Yes	732 (71.9)
No	286 (28.1)
Fluid intake (ml/day)	
≤2500	546 (53.6)
>2500	472 (46.4)
Exercise	
Yes	397 (39.0)
No	621 (61.0)
Waist-to-hip ratio (WHR)	0.9 ± 0.1
Urinary tract infection history ^a^	
Yes	93 (9.1)
No	925 (90.9)
Charlson comorbidity index (CCI)	1.7 ± 1.0
Diabetes mellitus duration (years)	9.0 ± 7.4
Diabetic peripheral neuropathy or retinopathy	
Yes	533 (52.4)
No	485 (47.6)
overactive bladder	
No overactive bladder	878 (86.3)
Dry overactive bladder	60 (5.9)
Wet overactive bladder	80 (7.9)

*SD* standard deviation, *RMB* Chinese Yuan, *USD* United States dollar

^a^Patients with type 2 diabetes who had a urinary tract infection at least one month before the survey

The unhealthiest toileting behavior among patients with diabetes was premature voiding, followed by place preference for voiding, delayed voiding, and straining to void (Table 2). Premature voiding and straining to void were significantly unhealthier in patients with overactive bladder than in those without overactive bladder, as indicated by Student’s *t*-tests. As shown in Table 3, about half of the patients often or always emptied their bladders with little or no need to urinate before sleep (57%) or before leaving home (47%). Approximately 36% of the patients often or always avoided using toilets at someone else’s house. With regard to public toilets, 26% of the patients were often or always worried about the cleanness and 24% of the patients avoided using them. More than 15% of patients often or always waited more than 4 h to urinate at work, and 13% of the patients often or always pushed down or strained to finish emptying their bladder.

**Table 2:** Comparison of toileting behaviors between patients with diabetes with and without overactive bladder

Toileting behavior	Overall sample (*n* = 1018)	No OAB (*n* = 878)	OAB (*n* = 140)	*p* value
Premature voiding	2.41 ± 1.00	2.37 ± 0.98	2.68 ± 1.04	0.001
Place preference for voiding	2.18 ± 1.14	2.18 ± 1.14	2.14 ± 1.13	0.710
Delayed voiding	2.06 ± 0.74	2.05 ± 0.73	2.15 ± 0.77	0.142
Straining to void	1.49 ± 0.88	1.45 ± 0.84	1.74 ± 1.04	0.002

The numbers are expressed as mean ± standard deviation

*OAB* overactive bladder

**Table 3:** Toileting behaviors among patients with type 2 diabetes mellitus (*n* = 1018)

Behavior	Never/Rarely	Sometimes	Often/Always
	*n* (%)	*n* (%)	*n* (%)
Premature voiding			
Empty bladder with little or no need to urinate before sleep	348 (34.2)	89 (8.7)	581 (57.1)
Empty bladder with little or no need to urinate before leaving home	412 (40.5)	131 (12.9)	475 (46.7)
Empty bladder with little or no need to urinate “just in case”	701 (68.9)	132 (13.0)	185 (18.2)
Empty bladder with little or no need to urinate at home	938 (92.1)	54 (5.3)	26 (2.6)
Place preference for voiding			
Avoid using toilets at someone else’s house	588 (57.8)	63 (6.2)	367 (36.1)
Worry about cleanness of public toilets	650 (63.9)	104 (10.2)	264 (25.9)
Avoid using public toilets	690 (67.8)	82 (8.1)	246 (24.2)
Hold urine until getting home	705 (69.3)	164 (16.1)	149 (14.6)
Delay voiding			
Wait too long (>4 h) to urinate at work	690 (67.8)	172 (16.9)	156 (15.3)
Delay emptying bladder when busy	572 (56.2)	326 (32.0)	120 (11.8)
Wait to empty bladder until unable to hold	788 (77.4)	152 (14.9)	78 (7.7)
Straining to void			
Push down or strain to finish emptying bladder	774 (76.0)	108 (10.6)	136 (13.4)
Push down or strain to begin urinating	883 (86.7)	64 (6.3)	71 (7.0)
Push down or strain to keep urine flowing	885 (86.9)	68 (6.7)	65 (6.4)
Push down or strain to empty bladder more quickly	880 (86.4)	80 (7.9)	58 (5.7)

After adjusting for control variables, positive associations between unhealthy toileting behaviors (premature voiding and staining to void) and overactive bladder were found among patients with diabetes (Table 4). The more the patients empty their bladder with little or no need to void and pushed down or strained to void, the greater the likelihood of having overactive bladder. There were no significant associations between toileting behaviors (place preference for voiding and delayed voiding) and overactive bladder. In addition, age, waist-to-hip ratio, CCI, and diabetic peripheral neuropathy or retinopathy were positively associated with overactive bladder, while female and married patients with diabetes were less likely to have overactive bladder.

**Table 4:** The relationships between toileting behaviors and overactive bladder among patients with type 2 diabetes mellitus (*n* = 1018)

Variables	OR (95% CI)	SE	*p* value
Toileting behavior			
Premature voiding	1.286 (1.048–1.579)	0.135	0.016
Straining to void	1.243 (1.026–1.506)	0.122	0.026
Delayed voiding	1.259 (0.969–1.636)	0.168	0.084
Place preference for voiding	0.883 (0.735–1.062)	0.083	0.188
Age	1.027 (1.006–1.049)	0.011	0.013
Sex (female vs. male)	0.484 (0.274–0.854)	0.140	0.012
Race/Ethnicity (Han vs. other)	4.624 (0.979–21.832)	3.662	0.053
Education (ref. = elementary school or lower)			
Middle school	0.831 (0.471–1.466)	0.241	0.522
High school	0.638 (0.360–1.130)	0.186	0.124
College or higher	0.584 (0.308–1.108)	0.191	0.100
Marital status (married vs. unmarried)	0.513 (0.285–0.922)	0.154	0.026
Living area (urban vs. rural)	1.219 (0.706–2.107)	0.340	0.477
Income (>3000 vs. ≤3000)	0.807 (0.523–1.245)	0.178	0.331
Smoker	1.105 (0.659–1.855)	0.292	0.705
Alcohol drinker	0.638 (0.379–1.073)	0.169	0.090
Tea drinker	1.215 (0.781–1.890)	0.274	0.388
Fluid intake (>2500 vs. ≤2500)	1.070 (0.726–1.575)	0.211	0.734
Exercise	0.823 (0.549–1.233)	0.170	0.345
Waist-to-hip ratio (WHR) ^a^	1.266 (1.033–1.552)	0.131	0.023
Urinary tract infection history ^b^	0.966 (0.496–1.880)	0.328	0.919
Charlson comorbidity index (CCI)	1.183 (1.001–1.397)	0.101	0.049
Diabetes mellitus duration	0.984 (0.955–1.013)	0.015	0.270
Diabetic peripheral neuropathy or retinopathy	1.506 (1.002–2.264)	0.313	0.049

*OR* odds ratio, *CI* confidence interval, *SE* standard error

^a^Standardized waist-to-hip ratio (WHR) was used in the analysis

^b^Patients with type 2 diabetes who had a urinary tract infection at least one month before the survey

## Discussion

A large sample survey of patients with type 2 diabetes was conducted to identify unhealthy toileting behaviors and further investigate their relationships with overactive bladder. The present study adds to the small body of work that extends research focused on only one or some aspects of toileting behavior to comprehensively examine it using a valid instrument. We found that the prevalence of overactive bladder in patients with diabetes was nearly 14%, which is more than twofold higher than that in the general population in China [3]. Moreover, the more the patients with diabetes engage in unhealthy toileting behaviors (e.g., premature voiding and straining to void), the greater the likelihood of having overactive bladder. Thus, our findings provide significant implications in the prevention, alleviation, and management of overactive bladder symptoms during diabetes care.

The findings indicated that the most common unhealthy toileting behavior among patients with diabetes was premature voiding. Approximately half of them often or always emptied their bladder with little or no need to void before sleep or before leaving home. Moreover, premature voiding was significantly associated with overactive bladder in patients with diabetes, which is in line with the finding by Wan et al., who found a positive association between premature voiding and lower urinary tract symptoms in female nurses [15]. In addition, too frequent voiding with little or no need to urinate may have no harmful effects in the short term; however, engaging in this behavior leads to increased bladder sensitivity to lower volumes of urine, reduced bladder capacity, and eventually detrusor instability in the long term [8, 9].

We also found that straining to void, although the least common unhealthy toileting behavior, had a positive association with overactive bladder in patients with type 2 diabetes. This finding confirms that from a study of female nurses with regard to straining to void and lower urinary tract symptoms [15]. Pushing down or straining was often or always employed by patients with diabetes to begin urinating (7.0%), keep urine flowing (6.4%), empty bladder more quickly (5.7%), and finish emptying bladder (13.4%). Abdominal straining to void is considered unhealthy by researchers because of its association with poor outcomes, such as fecal and/or urinary incontinence [34], dysfunctional voiding [13], and prolonged postoperative catheterization [35]. Pauwels et al. indicated that if straining was used to begin voiding before the initiation of the micturition reflex and as the only way to empty bladder, voiding problems and incontinence are more likely to occur [11].

In this study, delayed voiding and place preference for voiding were not significantly associated with overactive bladder in patients with diabetes; the patients with overactive bladder had almost the same scores for the two unhealthy toileting behaviors as those of patients without overactive bladder. Nevertheless, delayed voiding and place preference for voiding were common in patients with diabetes. Moreover, suppressing the urge to void may have no detrimental effect in the short term; however, long-term suppression may threaten bladder’s health [14]. Avoiding using public toilets or toilets at someone else’s house may lead to premature or delayed voiding [36]. Thus, educating patients to adopt healthy toileting behaviors to improve and maintain their optimal bladder health is essential.

Besides unhealthy toileting behaviors, we found another significant modifiable factor associated with overactive bladder in patients with diabetes, i.e., waist-to-hip ratio. Waist-to-hip ratio, as a measure of obesity or health, has been shown to be a better predictor of cardiovascular disease [37], diabetes [38], and hypercholesterolemia [39] compared with body mass index and waist circumstance. Our finding is similar to studies that showed obesity to be a risk factor of overactive bladder and weight loss to be related to improvements in incontinence status [20, 40]. Weight loss has a beneficial effect on general health and quality of life; thus, it should be encouraged universally. In addition, diabetic peripheral neuropathy or retinopathy, which is the consequence of poor blood glucose control, was found to be associated with overactive bladder. Similarly, Chiu et al. showed that the increased risk of overactive bladder in patients with diabetes could be attributed to higher glycosylated hemoglobin levels [19]. Furthermore, overactive bladder may also result from a number of conditions or comorbidities, such as hypertension, heart failure, cerebrovascular disease, dementia, and renal diseases [26], which supports our result that higher CCI was associated with increased risk of overactive bladder. Our results are consistent with a previous report that overactive bladder increased with age and was more prevalent in men than in women [20].

This study has several limitations. First, we recruited patients in one hospital using a convenience sampling method. Thus, the generalizability of our findings may be limited. To address this, we surveyed a large sample of 1025 patients with diabetes with <1% missing data. Second, because of the cross-sectional design of the survey, a causal relationship between unhealthy toileting behaviors and overactive bladder could not be established. Moreover, in cross-sectional studies, associations could be reversed. For example, patients might adopt certain toileting behaviors to cope with overactive bladder symptoms. However, this may not be the case in our study because we surveyed unhealthy toileting behaviors and later overactive bladder symptoms. Exploring toileting behavior changes after having overactive bladder symptoms and determining whether these changes further worsen or relieve overactive bladder symptoms would be interesting. Third, our findings may be influenced by uncontrolled confounders, such as constipation, benign prostatic hyperplasia, and vaginal or cesarean delivery. Future studies should include these variables in the survey and explore whether the associations between toileting behaviors and overactive bladder vary by gender.

## Conclusions

Overactive bladder in patients with type 2 diabetes was more than twofold higher than that in the general population. Thus, overactive bladder is not just an inconsequential condition for patients with diabetes. Furthermore, in patients with diabetes, preserving bladder health is particularly necessary and unhealthy toileting behaviors, especially premature voiding and straining to void, may contribute to the onset or worsening of overactive bladder. Identification and awareness of modifiable behavioral factors during diabetes care is an essential component of primary prevention, alleviation, and management of overactive bladder symptoms.

## Abbreviations

CCI: Charlson comorbidity index
OAB: Overactive bladder
OABSS: Overactive Bladder Symptom Score
TB-WEB: Toileting Behaviors-Women’s Elimination Behavior
